# Multi-level analyses of distance education capacity, faculty members’ adaptation, and indicators of student satisfaction in higher education during COVID-19 pandemic

**DOI:** 10.1186/s41239-021-00291-w

**Published:** 2021-10-12

**Authors:** Engin Karadag, Ahmet Su, Hatice Ergin-Kocaturk

**Affiliations:** 1 Faculty of Education, Akdeniz University, Kampus, 07070 Antalya, Turkey; 2 University of Toronto, Ontario Institute for Studies in Education, 252 Bloor Street West, Toronto, ON M5S 1V6 Canada; 3 Hasan Ali Yücel Faculty of Education, Istanbul University-Cerrahpaşa, Büyükçekmece Yerleşkesi, 34500 Istanbul, Turkey

**Keywords:** Student satisfaction COVID-19, Distance education capacity, Faculty members’ adaptation, Multi-level analyses

## Abstract

COVID-19 pandemic triggered distance education in higher education. Decisions such as isolation, social distancing and quarantine made by countries unexpectedly and suddenly forced face-to-face education to change to distance education within days. All academics around the world had to move online overnight. All the educational and academic activities in higher education (courses, exams, meetings, etc.) had to be conducted online in a few days. Based on these changes, this study aimed to analyze the relationships among student, faculty (adaptations of faculty members to distance education) and institutional (distance learning capacities of the universities) variables that affected satisfaction of the students related to distance education in higher education institutions in Turkey during COVID-19 pandemic using hierarchical linear modeling (HLM). The study group included 14,962 students and 3631 academics from 30 universities. The results showed that universities with higher distance education capacities got higher satisfaction scores. HLM analysis showed that 43% of the variation in satisfaction scores resulted from universities. The second HLM analysis showed that 44% of the overall satisfaction score variance of the students could be explained by the factors of university features (Level 2: distance education capacity and acceptance and use of distance education systems of faculty members). Thus, it was determined that 44% of the university factor calculated as 43% in Model 1 (which is calculated within students’ general satisfaction scores) resulted from the distance education capacity and the acceptance and use of distance education systems of faculty members. The findings of this study provide insights to improve distance education by stakeholders of higher education institutions.

## Introduction

On December 31st, 2019, China reported the discovery of a new type of coronavirus (SARS-CoV-2) pneumonia-like infection to the World Health Organization (WHO) in Wuhan, which caused serious illnesses and death (Yuan et al., 2020). By January 2020, the fact that the COVID-19 infection became a pandemic affecting more than 160 countries in a few weeks left the whole world to confront with a global problem. Since the coronavirus was spreading very quickly and was lethally dangerous in certain age groups and/or people with pre-existing medical conditions, the whole world took extensive measures such as the rapid closure of many workplaces and educational institutions following the spread of the virus. Many countries including Turkey decided to shut down K-12 schools and universities temporarily and continue educational activities through distance education. Higher education institutions, academic staff and students tried to adapt to this mandatory decision in a short time (Huang et al., 2020a, b). In this process, universities with reliable infrastructure continued through distance education systems, completed the 2019–2020 Spring semester, and planned to complete 2020–2021 Fall and Spring semesters in this way.

Audrey Azoulay, the Director-General of UNESCO (United Nations Educational, Scientific and Cultural Organization) stated that “We entered a region without a map, that is, the borders have been crossed.”, referring to the distance education (Huang, et al., 2020a, b). As the educational, scientific, and cultural organization of United Nations, UNESCO’s publications, and guidance gain importance in the times of such global crises. In this process, it was emphasized that all countries should work together to find high-tech, low-tech and non-technology solutions to ensure the continuity of teaching and learning (Huang, et al., 2020a, b). Tamrat and Teferra (2020) stated that universities should focus on the long-term higher education plans while concurrently working on the crisis management of the COVID-19 pandemic distance education. However, according to Tamrat and Teferra (2020) African higher education institutions were late to act both regionally and nationally. The report published by the OECD (2020) revealed that during the COVID-19 pandemic, educators and administrators of educational institutions had insufficiencies in areas such as distance education, composing online classes, and supporting students. In addition, higher education institutions and their stakeholders around the world constituted one of the groups most affected by the COVID-19 pandemic (Crawford et al., 2020).

In more than 85% of all countries, schools were shut down completely or gradually, meaning that schools have been no longer accessible for more than 1.6 billion students (on April 10, 2020). According to the study conducted with high school principals in 82 countries participating in the International Student Assessment Program (PISA), the rate of students and teachers using those platforms is between 35 and 70% even in schools with an effective online learning platform (The World Bank Education Global Practice, 2020a, b). The rates and the cases were similar to the above-mentioned World Bank data in Turkish higher education institutions. Although the Council of Higher Education (COHE) allowed up to 30% of the courses in undergraduate and graduate programs to be delivered through distance education with various regulations and directives, this rate did not exceed 5% in universities prior to the COVID-19 pandemic. The practices and encouragement of the COHE are very important indicators of supporting education with online procedures in the days when access to educational institutions is limited. However, it should be considered that the situation is much worse in middle and low-income countries where the rate of access to the internet is generally less than 50% and the rate of students who do not have any tools that enable online learning at home is high. For this reason, some countries turned to low-tech options such as television and radio to increase access to distance learning significantly (The World Bank Education Global Practice, 2020a). With the change from in-person education to distance education to stop the spread of the coronavirus, students who were living in regions with low-quality internet access and/or with low internet quotas were affected seriously. Some countries tried to overcome this problem by providing free internet service to students (Tamrat & Teferra, 2020).

Along with the whole world, Turkey had to resort to distance education. First, as of March 16, 2020, in-person education was suspended for three weeks in all primary, secondary and high schools, and higher education institutions. In addition, the COHE (YÖK, 2020a) decided to suspend the in-person classes for the associate and undergraduate students during this three-week period. As the number of COVID-19 cases increased rapidly, it was understood that the pandemic would last longer than expected. COHE announced that educational activities would be maintained through distance education, open education, and digital teaching tools and techniques on March 26, 2020 (YÖK, 2020b).

Turkey had an opportunity to take necessary precautions and get ready for distance education as the first cases were detected later than many European countries. In addition to this, thanks to the distance education infrastructure and experience, universities were expected to manage this crisis by putting this previously built capacity into action. In this respect, the pandemic has shown the competence levels of universities in the Turkish higher education system in several areas such as the management of distance education, digital tools and technical infrastructure competence, proficiency of instructors, and the quality of teaching materials. The evaluation of student experience in higher education is linked to the evaluation of the services and facilities at universities (Lin et al., 2020). Distance education which brings together many concepts such as digital learning, e-learning and mobile learning (Basak et al., 2018) has become popular in US higher education recently (Allen and Seaman, 2014). Similarly, distance education capacity plays an important role in the quality of distance education and student satisfaction. However, education literature needs more studies on student satisfaction in distance education.

However, many challenges were encountered during the COVID-19 pandemic. While some of the shortcoming’s stem from the transition to the distance education system in a short time, a significant part of them stemmed from the inadequacies of the universities’ capacities, the lack of adaptation of the faculty members and also from the students’ lack of the necessary technologic tools. In this context, in-depth examination of distance education in higher education will contribute to the development of the higher education system, to receive feedback on the education services provided and to increase the quality of education services, as well as to draw roadmaps on how to continue higher education in COVID-19 and different pandemic and emergency situations. In addition, the evaluation of distance education in universities during the COVID-19 pandemic will shape the level of technology integration of lecturers and students’ expectations and experiences. Based on this background, the main purpose of this study is “to determine and evaluate the distance education capacities of universities, acceptance and use of distance education systems of faculty members and the satisfactions levels of students”. The following sections gives a detailed literature review and links this background to the research questions of the study.

## Conceptual framework and the research questions

Satisfaction is a structure related to the evaluation of perceived inconsistencies between expectations for a product or service and the results (feeling and feedback) after the product or service was used (Oliver, 1981). Elliot and Healy (2001) define the concept of student satisfaction as an attitude resulting from the evaluation of experiences, services, and opportunities (Lin et al., 2020). Student satisfaction has become an important target for higher education (Guo, 2016). In recent years, researchers began to approach student satisfaction as a way of evaluating the overall performance of universities (Martirosyan, 2015). In addition, the strategic and economic importance of satisfaction studies in higher education, in various research areas such as state universities (Gruber et al., 2010), private institutions (Arif et al., 2013), e-learning programs (Sun et al., 2008), graduate programs (Carter, 2009), and extension programs (Marzo-Navarro et al., 2005) attracted the attention of academics and management.

Questionnaires are widely used to evaluate the satisfaction levels of students (Yorke, 2009). The oldest of those is the College Student Satisfaction Questionnaire (CCSQ), which was developed by Starr et al., (1971) and includes five indicators covering all aspects of the university life of students in the USA. The most widely used survey for student satisfaction in the UK is the National Student Survey (NSS) conducted by Ipsos MORI (Thiel, 2019). NSS consists of 27 questions with a 5-point Likert-type scale ranging from “strongly agree” to “strongly disagree”, which is administered to all senior undergraduate students. Course Experience Questionnaire (CEQ) (Griffin et al., 2003; McInnis et al., 2001) and Student Experience Survey (SES) (Morgan et al., 2018) used in Australia focus on different aspects of the student experience that may be measurable and potentially related to learning and development outcomes. CEQ measures five aspects of student experience: skill development, student engagement, teaching quality, student support, and learning resources. The theoretical framework of the study was based on this satisfaction and student experience literature. The data collection tools to evaluate student satisfaction were prepared based on the CEQ (McInnis et al., 2001) and NSS (National Student Survey, 2020) in student satisfaction literature; however, the rapid changes during the COVID-19 pandemic necessitated a special approach and special items in the questionnaire.

The most commonly used measure of student satisfaction in Turkey is Turkey University Satisfaction Survey (TUSS) carried out by University Assessments & Research Laboratory (UniAR) established in 2016 (Karadağ & Yücel, 2020a). The survey used in the TUSS consists of 60 Likert-type scale questions with an answering scale ranging from 1–10 and is administered to undergraduate students in all grade levels of all universities in Turkey. TUSS focuses on six aspects of student satisfaction: satisfaction in the learning experience, satisfaction in the campus and campus life, academic support and interest, satisfaction with the management and operation of the institution, wealth of learning opportunities and resources, and personal development and career support. Results obtained from TUSS have a great impact on institutional reputation and good results are used for marketing and public relations purposes. One of the main functions of TUSS and similar national student satisfaction surveys is to provide prospective students with information that will help them choose their university (Canning, 2015).

The abundance of research on satisfaction in higher education institutions in recent years and the various methodological approaches used in previous studies make it difficult to choose among various options for measuring structures related to satisfaction. Moreover, there are an excessive number of questionnaire structures (premises and conclusions) which are associated with satisfaction in higher education (Sultan & Yin Wong, 2014). Although there are a significant number of publications on this subject, the results of these studies are complicated and they differ in statistical significance, direction and even size (Yavaş & Babakus, 2010). For example, there is a negative relationship between the content of a course and student satisfaction in China (Liu, 2012), while in Romania (Munteanu et al., 2010), United Arab Emirates (Wilkins & Stephens-Balakrishnan, 2013) and Armenia (Martirosyan, 2015) a positive relationship was found. Again, Ledden and Kalafatis (2010) and Clemes et al. (2008) found a high level (r > 0.67) correlation between satisfaction and recommendation intention in higher education institutions, while Athiyaman (1997) did not observe a relationship. In addition, it is documented in the relevant literature that when it is more difficult to evaluate the quality of educational services, the effect of expectations in the reaching satisfaction will be more difficult. (Anderson & Sullivan, 1993; Yi, 1993).

Another focus of the study was to investigate the distance education capacities of the universities. The comprehensive distance education and technology infrastructure standards were taken as the foundation to analyze the distance education capacities of Turkish universities (Bergeron & Fornero, 2018; Moore and Fodrey (2018). Piña (2018) also recommended assessing the infrastructure and capacity of the institution as a starting place to improve distance education, which resonated with the current study. Moore & Fodrey state that the critical components of the technological infrastructure are systems, objectives, personnel, and evaluation. Therefore, this study focused on analyzing the capacity of universities using this framework as a foundation. On the other hand, technology has changed the way educators teach and the way students learn because it has the potential to improve students’ learning experience (Glover et al., 2016). With the increasing use of mobile devices among students, particularly generation Y, traditional ways of providing learning materials through learning management systems are becoming less useful in creating effective learning environments, as they have limitations such as being less learner-centered and allowing only certain activities (Yasar & Adıgüzel, 2010). Although higher education institutions commonly use learning management systems (LMS) to facilitate student learning, most of the teacher-centered higher education institutions fail to offer LMS’s along with Web 2.0 features such as effective distance education and dynamic content (Anderson & Dron, 2017). Universities have not actively embraced distance education systems despite the benefits gained from the use of these modes of instruction. Based on this background, the current study was conducted to better understand distance education satisfaction of students in the COVID-19 pandemic. For this reason, the research question (RQ) below was examined in this study:

### RQ_1_

How is the student satisfaction with distance education?

The main factors affecting student satisfaction are the quality of teaching in the classroom, the quality of feedback given to students, and student-faculty relations in the classroom (Hill et al., 2003; Siming, et al., 2015). Students are more likely to be satisfied if faculty members can effectively involve students in teaching activities. The more engaged students are in learning, the more likely they are to learn and be satisfied (Jankowski, 2017). In summary, since the success of students is largely dependent on the teaching attitude of the instructor and the effectiveness of teaching materials and technology, it is very important that faculty members adopt teaching practices that create such learning environments. For this reason, the research questions below were tested:

### RQ_2a_

How is the effect of the distance education capacities of universities on students’ satisfaction?

### RQ_2b_

How does the level of acceptance and use of distance education systems by faculty members affect students’ satisfaction?

### RQ_3_

How is the students’ attitude towards distance education?

## Methodology

### Participants

The population of the study consists of 2,348,535 undergraduate students studying at 195 universities in Turkey and 150,343 academics who work in these universities. In addition, the distance education capacities of universities and the acceptance and use of distance education systems by faculty members were discussed as a factor affecting student satisfaction. While determining the universities to collect the data, the universities were first divided into two categories as state and foundation (run by non-profit organizations) universities, then divided into 10 subcategories according to the number of students. 2 state and 1 foundation university were selected from each sub-category; thus 30 study universities were determined. After this, the study employed email list sampling, and an email explaining the aim of the study was sent to the academics and students. The academics and students who volunteered to participate in the study were sent the data collections tools of the study. The data were collected from 14,962 undergraduate students and 3,631 academics who voluntarily participated in the study. As the confidence interval in the study was accepted as 0.98 and the margin of error as 0.02, the minimum sample to represent a total of 2,348,535 undergraduate students was 3310; and similarly, the minimum sample to represent a total of 150,343 academics was 3310 as well (YÖK, 2020e, Hamburg, 1985). In this respect, the samples consisting of 14,962 students and 3,631 academics are sufficient to represent the population. Detailed information about the participants is presented in Table 1.

**Table 1:** Cronbach’s Alpha reliability coefficients of the scales

Akdeniz Distance Education Satisfaction Scale	Item number	α
1. Satisfaction with Council of Higher Education	3	0.95
2. Satisfaction with University and Faculty Management	3	0.91
3. Satisfaction with Digital Content/Instruction Material	8	0.96
4. Satisfaction with Faculty Members	5	0.94
5. Satisfaction with Technical Infrastructure	5	0.94
6. Satisfaction with Learning/Teaching Process	11	0.96
7. Satisfaction with Assessment-Evaluation Process	2	0.79
Total	**37**	**0.98**

### Data collection instruments

#### Distance education capacity assessment form

Data on distance education capacities of the universities were obtained using the “Distance Education Capacity Assessment Form” developed within the scope of the study. The evaluation form was developed based on the contents of ‘Distance Education and Technology Infrastructure’ created by Bergeron and Fornero (2018) and Moore and Fodrey (2018). The first part, which consists of 46 items with a structured open-ended form, consists of six sub-dimensions: (*a*) ‘Human Resources Capacity’, (*b*) ‘Hardware Infrastructure Capacity’, (*c*) ‘Software Infrastructure Capacity, (*d*) Content Production Capacity’, (*e*) ‘Exam Infrastructure Capacity and (*f*)’ Budget ‘.

#### Akdeniz distance education satisfaction scale

The 7-factor scale is comprised of 37 items. The scale aims to measure students’ satisfaction with distance education during the COVID-19 pandemic. High scores indicate a higher level of satisfaction. The questionnaire items are structured as a 10-point Likert scale ranging from 1 (not very satisfied) to 10 (very satisfied). In the current study, the internal consistency coefficient (Cronbach Alpha) the scale is between 0.79 and 0.96 (Table 1). The following are example items from the scale:

- I am satisfied with my university’s distance education preparation.
- I am satisfied with the teaching of digital content/teaching materials.
- I am satisfied with the sound and image quality of the distance education system.
- I am satisfied with the efficiency level of distance education lessons.

## Acceptance and Use of Distance Education Systems Scale

The scale consists of 25 items and has a seven-factor structure. The scale items were prepared based on ‘Extending the Unified Theory of Acceptance and Use of Technology’ developed by Venkatesh, Thong and Xu (2012) to determine the acceptance and use of distance education systems by faculty members. High scores indicate higher levels of acceptance and use of distance education systems. The questionnaire items were structured as a 10-point Likert scale ranging from 1 (totally disagree) to 10 (totally agree). In the present study, the internal consistency of the data of the scale is between 0.76 and 0.98 (Table 2). The following are example items from the scale:

- Using the distance education system increases my productivity.
- Learning to use the distance education system is very easy for me.
- I have the necessary resources to use the distance education system.
- From the efficiency level of distance education lessons.
- The use of the distance education system is a have become a habit for me.

**Table 2:** Cronbach’s Alpha reliability coefficients of the scales

Acceptance and use of distance education systems scale		
1. Performance expectation	4	0.93
2. Effort expectation	4	0.85
3. Social effect	3	0.91
4. Facilitating factors	4	0.86
5. Hedonic motivation	3	0.97
6. Habit	4	0.76
7. Behavioral intention	3	0.98
Total	**25**	**0.89**

### Procedure

At the beginning of the study, the “Social and Human Sciences Scientific Research and Publication Ethics Committee” approved the study protocol (Akdeniz University Decision No. 136, 18.06.2020). The participants were contacted with a data collection package containing demographic questions and scale items. Firstly, the purpose of the study was explained to the participants, informed consent forms were collected, and the participants were informed about the confidentiality of the data, voluntariness, and anonymity of participation. It took about 30 min for the participants to complete the research package.

**Fig. 1:**
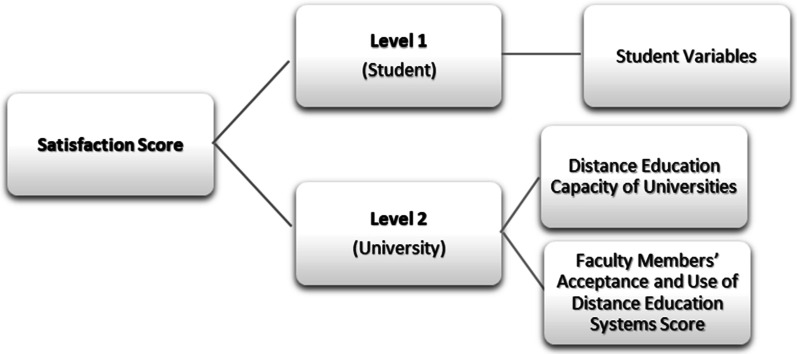
Conceptual framework of the study

Two-level hierarchical linear modeling (HLM) analysis was used to determine the factors affecting students’ satisfaction in Hierarchical Linear Modeling (HLM, ver. 8). Two-level HLM was preferred for analysis because the data were collected from different layers (student, faculty, and university) and the dataset reflects an intertwined and hierarchical structure. The data were obtained from the university and faculty members layers at the macro level (Level 2) and from the student layer at the micro level (Level 1) (Fig. 1). Considering education hierarchically, individual students cluster in universities (Bryk & Raudenbush, 1992; Goldstein, 1995; Hox, 2010). Therefore, an estimate related to a student is influenced by the student’s variables, the qualifications of the faculty member and university at he or she is studying. Ignoring such hierarchical relationships makes the influence of the group on individual predictions overlooked (Goldstein, 1999). The main purpose of this study is to see the difference between universities, not between the faculties. For this reason, the faculties at which students’ study were not taken as an analysis level in this study. Level 1, the student basic model is as follows:

1 $$\left( Y \right)_{{ij}} = \beta _{{0j}} + r_{{ij}}$$

where (*Y*)_*ij*_ is satisfaction score for student *i* in university *j*. *β*_*0j*_ is the mean score of student satisfaction in university *j* and *r*_*ij*_ is random effect at student level. Level 2 basic model is as follows:

2 $$\beta_{0j} = \gamma_{00} + u_{0j}.$$

Here *γ*_*00*_, overall average (or intersection) of student satisfaction between universities) and *u*_*0j*_ is random effect at university level. These formulas were expanded with the independent variables used in the study. Students’ demographics (sex, grade level, etc.) were included in the Level 1 model as separate independent variables, and the distance education capacities of universities and faculty members’ acceptance and use scores of distance education systems were included separately in the Level 2 model.

## Findings

### Descriptive analysis

**Graph 1:**
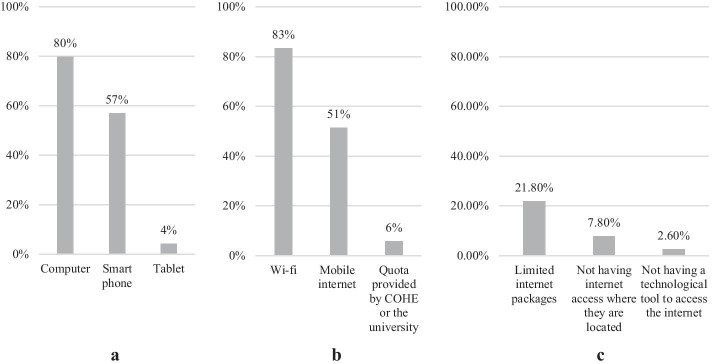
Tools, internet access methods of participant students and the reasons for missing their distance education classes

Technological tools used by students to attend their distance education classes were presented below (Graph 1a). According to the results, 11,954 (79.9%) of the students reported that they attended the distance education classes with their computers; 8525 (57.0%) with their smartphones and 633 (4.2%) with their tablets. On the other hand, 2900 of the students (19.4%) reported they did not have a computer to attend their online courses. Almost all these students attended their classes on their smartphones.

Internet access providers that students use to attend their distance education lessons were presented below (Graph 1b.). 12,460 (83.3%) of the students reported that they attended the distance education lessons using Wi-Fi; 7684 (51.4%) mobile internet and 866 (5.8%) the quota provided by COHE or the university. Additionally, 2353 of them (15.7%) had no internet access.

The reasons for students’ missing distance education lessons were presented below (Graph 1c). 3,268 (21.8%) of the students reported that they missed distance education lessons from time to time due to limited internet packages; 1,187 (7.9%) due to not having internet access where they are located, and 384 (2.6%) due to not having a technological tool to access the internet.

The details of students’ satisfaction with the distance education are presented below (Table 3). According to the findings, only “Higher Education Council satisfaction” (*M* = 7.7, *SD* = 1.9) score was at a very good level. On the other hand, the score of students’ satisfactions with the measurement-evaluation process (*M* = 5.5, *SD* = 3.1) was at medium-lower level. Similarly, students’ “digital content/teaching material satisfaction” (*M* = 6.1, *SD* = 2.8), “learning/teaching process satisfaction” (*M* = 6.1, *SD* = 2.7), “instructor satisfaction” (*M* = 6.0, *SD* = 2.9), “technical infrastructure satisfaction” (*M* = 5.9, *SD* = 2.9) and “university and faculty management satisfaction” (*M* = 5.7, *SD* = 3.0) scores were found to be moderate. The correlation coefficients of the relationships among the variables were presented below (Table 3). Results showed that there was a positive and significant correlation between the variable scores. The highest correlation was between “digital content/teaching material satisfaction” score and “learning/teaching process satisfaction” score (*r* = 0.90), the lowest correlation was between “Higher Education Council satisfaction” score and “instructor satisfaction” score (*r* = 0.56). In summary, the findings relating to RQ_1_ indicated that the satisfaction with distant education was low.

**Table 3:** The correlation coefficients of the variables

	***M***	***SD***	**1**	**2**	**3**	**4**	**5**	**6**	**7**	**8**
**1. **Satisfaction with council of higher education	7.7	1.9	-							
**2. ** Satisfaction with university and faculty management	5.7	3.0	0.65*	-						
**3.** Satisfaction with digital content/instruction material	6.1	2.8	0.64*	0.82*	-					
**4.** Satisfaction with faculty members	6.0	2.9	0.56*	0.80*	0.82*	-				
**5.** Satisfaction with technical infrastructure	5.9	2.9	0.67*	0.78*	0.87*	0.78*	-			
**6.** Satisfaction with learning/teaching process	6.1	2.7	0.64*	0.79*	0.90*	0.87*	0.86*	-		
**7.** Satisfaction with assessment-evaluation process	5.5	3.1	0.62*	0.73*	0.80*	0.74*	0.80*	0.83*	-	
**Total**	**6.1**	**2.5**	0.71*	0.87*	0.95*	0.90*	0.92*	0.96*	0.86*	**-**

*p < 0.001

### The multi-level analysis of the factors affecting student satisfaction

The effect of student-related factors (Level 1), the distance education capacity levels of universities and the faculty’s acceptance and use of distance education systems (Level 2) on students’ overall satisfaction was examined through two-level HLM. In the first level, universities were classified as “low-level capacity”, “medium-level capacity” and “high-level capacity” based on their distance education capacity. In the second level, the mean scores of the distance education acceptance and use of the faculty members were calculated for each university and accepted as the university score.

#### Change of scores between universities

In the first level of the HLM analysis, an unconstrained model (Model 1) was applied to the data set to see whether the variability in students’ overall satisfaction scores was associated with universities. The results showed that universities differed significantly in terms of the students’ overall satisfaction scores (Coefficient = 6.17, *Sh* = 0.12, *sd* = 29, *t* = 51.24, *p* < 0.001). In addition, the reliability coefficient of the application was 0.919. Intraclass correlation coefficient was calculated for Model 1 and it was seen that the difference in overall satisfaction score resulted from universities (*df* = 29, *X*^*2*^ = 628.43, *p* < 0.001). Intraclass correlation coefficients were used to determine the variance ratio resulting from the second level (Eq. 3). The variance of Level 1 is represented as ${\sigma }^{2}$, and the variance of Level 2 is represented as ${\tau }_{00}$. Their significance tests are calculated using *x*^2^ test (De Leeuw & Kreft, 1986).

3 $${\varvec{\uprho}} =\frac{{\tau }_{00}}{{\tau }_{00}+{\sigma }^{2}}=\frac{4.479}{5.984+4.479}\hspace{0.17em}=\hspace{0.17em}0.43.$$

The ratio of this difference was calculated as 43%, and this result showed that 43% of the variance that can be explained by the general satisfaction scores was university-based (Level 2), and the remaining 57% was student-based (Level 1). In this respect, universities where students continued their education made a difference in their general satisfaction scores. In other words, there were significant differences between universities in terms of students’ general satisfaction.

### Effect of student characteristics on general satisfaction scores

Effect of student-related factors (Level 1) on students’ overall satisfaction was analyzed with the HLM’s random coefficient regression model (Model 2) (Table 4). The results showed that the variables which had a significant effect on students’ overall satisfaction scores were sex (*t* = 6.39, *p* < 0.001), grade level (*t* = 11.06, *p* < 0.001), distance education experience before the pandemic (*t* = − 4.68, *p* < 0.001) and teaching methods (*t* = − 19.99, *p* < 0.001). On the other hand, there were no significant effects of age (*t* = 0.08, *p* = 0.93) and faculty/college (*t* = − 1.08, *p* = 0.27) on general satisfaction scores. In addition, the reliability coefficient of the application was found to be 0.923.

**Table 4:** Student variables regression model coefficients with random coefficients

Fixed effect	Coefficient	Se	t	p
General satisfaction *β*_*0*_	6.574	0.150	43.65	< 0.001
Sex *γ*_*10*_	0.307	0.048	6.39	< 0.001
Age *γ*_*20*_	0.000	0.000	0.08	0.934
Grade level *γ*_*30*_	0.211	0.019	11.06	< 0.001
Faculty *γ*_*40*_	− 0.006	0.005	− 1.08	0.277
Distance education experience before pandemic *γ*_*50*_	− 0.219	0.046	− 4.68	< 0.001
Teaching methods *γ*_*60*_	− 0.655	0.032	− 19.99	< 0.001

HLM 6 takes the largest coded group as the reference in categorical variables. In this study, sex was coded as 1 = Female, 2 = Male. Accordingly, it is possible to comment that the overall satisfaction scores of male students also increased. It was observed that the grade levels of the students positively affected their general satisfaction scores, that is, the higher the grade level was, the more satisfied the students were. In the study, the variable of distance education experience before the pandemic was coded as 1 = yes, and 2 = no. Accordingly, the distance education experience of the students before the pandemic positively affected their overall satisfaction scores. Finally, the variables of how the lessons are taught were coded as 1 = synchronous, 2 = asynchronous, and 3 = homework submission. The findings indicated that synchronous method in distance education affected the general satisfaction scores of the students positively. The ratio of variables found to be significant among student characteristics (Level 1) to explain the variance of students’ general satisfaction score was calculated through the variance components obtained in the random intercept model (Eq. 4). The variance of Level 1 is represented as ${\sigma }^{2}$, the variance of Level 2 is represented as ${\tau }_{00}$, and the variance of the difference from the mean effect of the independent variable on the dependent variable is represented as ${\tau }_{11}$. Their significance tests are calculated using *X*^2^ test (Luke, 2004).

4 $$\text{R}^2=\frac{{\sigma }_{(ANOVA)}^{2}- {\sigma }_{(RKRM)}^{2} }{{\sigma }_{(ANOVA)}^{2}}=\frac{5.984-0.485}{5.984}= 0.91$$

The estimation result showed that Level 1 predictors explained the overall satisfaction score variance up to 91% (91% of 57%) (*X*^*2*^ = 664.935, *p* < 0.001) significantly. It was seen that more Level 1 variables were needed to explain the remaining 9% of the variance.

### Effects of the features of the universities on general satisfaction scores

The effect of the features of the universities (Level 2) on the overall satisfaction of students was examined with the result regression model of HLM (Model 3) (Table 5). The results showed that the university’s distance education capacity (*t* = − 1.08, *p* = 0.27) and faculty’s acceptance and use of distance education systems had a significant effect on students’ overall satisfaction scores. In the study, the distance education capacity variable of universities was coded as 1 = low level capacity, 2 = medium level capacity and 3 = high level capacity. Universities with high distance education capacity positively affected students’ overall satisfaction scores. In addition, it was observed that the acceptance and use of distance education systems by the faculty positively affected the overall satisfaction scores of the students, in other words, the more the faculty members’ adaptation to the distance education systems was, the more satisfied the students were. In addition, the reliability coefficient of the application was found to be 0.899. Using the variance component values, the ratio of explaining the variance of university characteristics (Level 2) was calculated (Eq. 5).

**Table 5:** University variables random coefficient regression model coefficients

Fixed effect	Coefficient	Se	t	p
General satisfaction *β*_*0*_	6.182	0.103	60.005	< 0.001
Distance education capacity *γ*_*01*_	0.258	0.148	4.98	< 0.001
Faculty’s acceptance and use of distance education systems *γ*_*02*_	0.256	0.118	5.14	< 0.001

5 $$\upbeta 0j \,explained \,variance \,ratio =\frac{ {\tau }_{00 \left(ANOVA\right)-{\tau }_{00 \left(Son\,Ort\,Old\,Model\right)}} }{{\tau }_{00 \left(ANOVA\right)}}=\frac{5.984-3.372}{5.984} = 0.44$$

As a result, it was observed that 44% of the variance in the general satisfaction score of the students was explained by the factors of university characteristics (Level 2: distance education capacity and the faculty’s acceptance and use of distance education systems) (*X*^*2*^ = 410.88374, *p* < 0.001). Thus, 44% of the university factor (calculated as 43% in Model 1) stemmed from the distance education capacity and the acceptance and use of distance education systems by faculty members. The analysis indicated that more Level 2 variables were needed to explain the remaining 56% of variance.

### The effects of the interactions between student and university features on general satisfaction scores

The effects of the interactions between Level 1 (student factors) and Level 2 (distance education capacity and the faculty’s acceptance and use of distance education systems) variables on students’ overall satisfaction scores were analyzed using the random intercept and slope model (Model 4) (Table 6). The results showed that the cross-interaction between sex and university characteristics positively affected the overall satisfaction scores (*t* = 6.79, *p* < 0.001). This means that the overall satisfaction scores of male students increased in universities where distance education capacity and academic staff adaptation were high. It was determined that the cross-interaction between grade level and university characteristics positively affected the general satisfaction scores (*t* = 11.80, *p* < 0.001). This means that the higher the grade levels of the students were, the higher their overall satisfaction scores were in universities with high level of distance education capacity and faculty adaptation.

**Table 6:** Intersection and slope model regression model coefficients

Fixed effect	Coefficient	Se	t	p
General satisfaction *β0*	6.180	0.108	57.00	< 0.001
Sex *x* level 2 variables *γ10*	0.330	0.048	6.79	< 0.001
Grade level *x* level 2 variables *γ20*	0.231	0.019	11.80	< 0.001
Distance education experience *x* level 2 variables *γ30*	− 0.242	0.047	− 5.07	< 0.001
Teaching methods *x* level 2 variables *γ40*	− 0.728	0.037	− 19.31	< 0.001

In addition, it was observed that the overall satisfaction scores of the students studying in universities with distance education experience, high distance education capacity and academic staff adaptation were significantly higher (*t* = − 5.07, *p* < 0.001). Similarly, in universities where distance education capacity and academic staff adaptation were high, as the number of courses offered synchronously increased, the overall satisfaction scores tended to increase (*t* = − 19.31, *p* < 0.001). In addition, other cross interactions in Model 4 were found to be insignificant. The variance ratio explained by the cross-interaction terms was calculated using the variance components value (Eq. 6).

6 $$\text{R}2 =\frac{ ({\tau }_{00 \left(RKRM\right)}- {\tau }_{00 (KEP\text{\c{C} }M)} }{{\tau }_{00 \left(RKRM\right)}}=\frac{5.662-1.382}{5.662}= 0.75$$

The results showed that cross-interactions between university (Level 2) and Level 1 factors created significant variation in overall satisfaction scores. However, these interactions only accounted for 75% of the change in the outcome variable. In summary, the findings relating to RQ_2a_ and RQ_2b_ indicated that satisfaction with distance education was affected by student features and university features.

### Views regarding distance education

Students’ general judgments towards distance education were described below (Table 7). The scores of the students views on “Distance education should be used as supportive of formal education.” (*M* = 6.1, *SD* = 3.5) was at medium level. Other judgments regarding distance education were at middle-low level. The view with lowest mean score was the following: “Distance education could be utilized in all courses in formal education.” (*M* = 4.0, *SD* = 3.6). This was followed by these views: “Distance education is as efficient as face-to-face education.” (*M* = 4.3, *SD* = 3.5) and “Distance education makes learning easier.” (*M* = 4.4, *SD* = 3.5). In addition, the mean scores of the following items were at medium–low level: “Distance education is an effective learning model.” (*M* = 4.4, *SD* = 3.5) and “Distance education system is useful.” (*M* = 5.2, *SD* = 3.6). In summary, findings indicated that students’ attitude towards distance education was low.

**Table 7:** Descriptive statistics of judgments regarding distance education

	***M***	***SD***
1. Distance education is as efficient as face-to-face education	4.3	3.5
2. Distance education makes learning easier	4.4	3.5
3. Distance education system is useful	5.2	3.6
4. Distance education is an effective learning model	4.5	3.5
5. Distance education should be used as supportive method to formal education	6.1	3.5
6. Distance education could be utilized in all courses in formal education	4.0	3.6

*p < 0.001

## Conclusion

Students’ satisfaction with the distance education processes carried out at universities during the COVID-19 pandemic was examined in this study. We found that students’ satisfaction levels were low. In addition, the distance education capacities of the universities and the acceptance and use of the distance education systems of the faculty members were found to have a significant positive effect on the overall satisfaction scores of the students. The following table shows a summary of the results relating to the research questions (Table 8).

**Table 8:** Summary of test results

	**Relations**	**RQs**
RQ_1_	Satisfaction with distance education	Low
RQ_2.a_	Student Features → General Satisfaction	Positive
RQ_2.b_	University Features → General Satisfaction	Positive
RQ_3_	Distance Education Judgment	Low

According to the results of this study, 80% of the students followed their distance education lessons from their computers, 57% from their smart phones and 4% from their tablets. In addition, one out of every five students (20%) did not have a computer. In the study, 83% of the students had Wi-Fi connection. This rate was 63% in a comprehensive study conducted by Karadağ and Yücel (2020b) on April 1–4, 2020. The reasons for students’ missing distance education courses were as follows: limited internet packages (20%), lack of internet access where they were located (8%), and no technological tools to access the internet (3%). The frequency of students who missed their lessons due to the lack of technological tools (computer, tablet, etc.) was lower than the study of Karadağ and Yücel (2020b). The comparative results indicate that, a significant portion of the students arranged Wi-Fi connection and technological tools during the 3-month period between these two studies. Another similar study conducted with 2781 students (Kırşehir Ahi Evran University, 2020) showed that 23% of the students could not attend online courses. Besides, together with the results of the previous studies, this study showed obviously that distance education additional cellular data quota provided by some of the universities and COHE (YÖK, 2020c) was a beneficial and to-the-point practice.

## Discussion

According to the findings of the current study, the results of the RQ_1._ show that the students were dissatisfied with distance education in the COVID-19 pandemic. With regard to the satisfaction dimensions, students were satisfied only with the decisions taken by the COHE during the pandemic. In contrast, dissatisfaction in other areas was expected. Even though the distance education and open course materials had a wide coverage and many universities claimed ensuring an effective transition to digitalization, there are studies indicating that this is not realistic (Karadağ & Yücel, 2020b). Effendi, Sugandini and Istanto (2020) argue that the COVID-19 pandemic is accelerating social media adaptation and digitalization. However, many studies on the COVID-19 pandemic, distance education and remote work processes reveal critical results. Kedraka and Kaltsidis (2020) state that there are no problems in terms of adaptation due to the age of students and their openness to technology, but the overall satisfaction with distance education processes is low for various reasons. Similarly, Allo (2020) found that despite receiving some positive feedback from students, many deficiencies and complaints regarding distance education satisfaction were expressed. He claimed that students in Indonesia found offline methods more effective in the earlier days of the pandemic and stated that learning management systems were used later. It also revealed that practices such as internet quotas constitute one of the major obstacles (Wargadinata et al., 2020). For this reason, the findings obtained in the study are compatible with the literature.

The study results on RQ_2a_ and RQ_2b_ indicated the distance education capacity of universities and the level of acceptance and use of distance education systems by faculty members had a positive effect on general satisfaction of the students. Similar results were reported in the literature, especially in studies examining the effect of distance education systems. Another study which used the same model (UTAUT) and examined the same variables as this study revealed that students’ technology acceptance and use and perceptions on the faculty’s technology competencies affected students’ distance education satisfaction (Alshare & Lane, 2011). DeBourgh (1999) stated that the lecturer and the method of teaching contributed to the explanation of the variance in student satisfaction in a program conducted interactively with various methods. In terms of teaching staff and teaching processes, it is reported that explicit expectations regarding the studies and the immediate response and feedback to students’ questions are directly related to student satisfaction. Kane et al. (2016) revealed that students’ satisfaction with their faculty members who participated in online education processes increased over time. In other words, as a faculty member continues her/his development within the distance education system, student satisfaction with this specific faculty member increases. In this case, it is accurate to say that as the faculty members gain more experience and manage their online education processes better, they provide higher satisfaction. Aman (2009), on the other hand, conducted an experimental study evaluating the change in student satisfaction over time when the faculty members gave feedback to each other in online education. As a result of the study, a significant increase in student satisfaction was found in the group where faculty members gave feedback by watching each other’s courses. Therefore, it can be stated that increasing the competence of faculty members in distance education through various methods will contribute to student satisfaction.

Also, the fact that the students had distance education experiences before the pandemic affected the students’ satisfaction positively. There are three main indicators of pre-pandemic distance education experience. The first is that the universities with a pre-pandemic distance education experience have a more advanced distance education capacity and experience. Secondly, the students at these universities are used to the distance education system, lesson preparation, exams, materials and most importantly, and they have the technological tools. Lastly, many faculty members and lecturer at these universities have distance education experience. The intersection of these three indicators helps the universities overcome the chaos caused by quick transition to distance education during Covid-19 pandemic and increases the level of satisfaction by accelerating the adaptation. Similarly, satisfaction is higher especially in universities and faculties where lessons are conducted synchronously. This result is also consistent with previous findings.

Various studies on distance education also reveal that experience with technological tools and internet self-efficacy positively affect the distance education experiences of students and thus their satisfaction (Kuo et al., 2013). In this context, Kuo et al. (2013) stated that students with high internet self-efficacy have a more advanced ability to search and access information. So, it will be beneficial for institutions to provide students with training and studies that will improve these skills and self-efficacy in order to improve students’ online experiences and satisfaction. Kırmızı (2015), on the other hand, evaluated the effects of students’ readiness in higher education on their satisfaction in an online program. The study revealed that there is a positive relationship between computer/internet self-efficacy and satisfaction. In their comprehensive study on online learning outcomes, Chu and Chu (2010) evaluated the relationship between various structures. In this study, it is revealed that peer support affected internet self-efficacy, and internet self-efficacy also positively affected learning outcomes. It is accurate to conclude that the mutual interaction between these structures increases students’ online learning satisfaction. Therefore, the fact that universities and students have distance education experience is seen as a factor that will contribute to students’ achieving more efficient results from distance education processes and their satisfaction.

The results of this study indicate clearly that Turkish universities were unsuccessful in managing distance education in COVID-19 pandemic. The main problems were inadequacies of the universities’ infrastructure, delay in adaptation of the faculty members, failure to evaluate and answer the problems of the students on time, failure to provide the necessary guidance at the right time, and the problems of students in accessing technological devices and the internet. We can infer from the results that universities are not prepared enough for the distance education and they have not been able to achieve the criteria determined by COHE. Based on these results and the inability to adapt to the distance education, it will not be accurate to expect the Turkish higher education system to be able to teach in digital environments (Karadağ & Yücel, 2020b). Lastly, the results on students’ judgements related to their distance education experiences give the signs of the future problems.

According to the results of the study, universities in Turkey and around the world should increase their distance education capacity without depending on any reason such as COVID-19 pandemic or another emergency, and faculty members should increase their digital competencies. As the learning environments change rapidly, planning focuses on this change and transition. The continuity of planning learning processes includes not only the technologies that the higher education institution will utilize to continue education, but also how students will return to campus after the emergency is over (International Baccalaureate Organization, 2020). For this reason, there is still further need for a multidimensional examination of the current distance education processes and data on the competencies and needs of the students to return to the campus at the end of the COVID-19 pandemic.

## Data Availability

The datasets generated and analyzed during the current study are available from the corresponding author on reasonable request.
